# *Elizabethkingia anophelis* MSU001 Isolated from *Anopheles stephensi*: Molecular Characterization and Comparative Genome Analysis

**DOI:** 10.3390/microorganisms12061079

**Published:** 2024-05-27

**Authors:** Shicheng Chen, Steven Pham, Nicolas Terrapon, Jochen Blom, Edward D. Walker

**Affiliations:** 1 Medical Laboratory Sciences Program, College of Health and Human Sciences, Northern Illinois University, DeKalb, IL 60115, USA; 2 Corewell Health William Beaumont University Hospital, Royal Oak, MI 48073, USA; sphamresearch@gmail.com; 3 Laboratoire Architecture et Fonction des Macromolécules Biologiques (AFMB), UMR7257 CNRS AMU, USC 1408 INRAE, 13009 Marseille, France; nicolas.terrapon@univ-amu.fr; 4 Bioinformatics and Systems Biology, Justus-Liebig University Giessen, 35392 Giessen, Germany; jochen.blom@computational.bio.uni-giessen.de; 5 Department of Microbiology, Genetics, and Immunology, Michigan State University, East Lansing, MI 48824, USA; walker@msu.edu

**Keywords:** *Elizabethkingia anopheles*, interaction, genome analysis, symbiotic traits

## Abstract

*Elizabethkingia anophelis* MSU001, isolated from *Anopheles stephensi* in the laboratory, was characterized by matrix-assisted laser desorption/ionization time of flight mass spectrometry (MALDI-ToF/MS), biochemical testing, and genome sequencing. Average nucleotide identity analysis revealed 99% identity with the type species *E. anophelis* R26. Phylogenetic placement showed that it formed a clade with other mosquito-associated strains and departed from a clade of clinical isolates. Comparative genome analyses further showed that it shared at least 98.6% of genes with mosquito-associated isolates (except *E. anophelis* As1), while it shared at most 88.8% of common genes with clinical isolates. Metabolites from MSU001 significantly inhibited growth of *E. coli* but not the mosquito gut symbionts *Serratia marcescens* and *Asaia* sp. W12. Insect-associated *E. anophelis* carried unique glycoside hydrolase (GH) and auxiliary activities (AAs) encoding genes distinct from those of clinical isolates, indicating their potential role in reshaping chitin structure and other components involved in larval development or formation of the peritrophic matrix. Like other *Elizabethkingia*, MSU001 also carried abundant genes encoding two-component system proteins (51), transcription factor proteins (188), and DNA-binding proteins (13). *E. anophelis* MSU001 contains a repertoire of antibiotic resistance genes and several virulence factors. Its potential for opportunistic infections in humans should be further evaluated prior to implementation as a paratransgenesis agent (by transgenesis of a symbiont of the vector).

## 1. Introduction

*Elizabethkingia anophelis* is an aerobic, non-fermenting, non-motile, and non-spore-forming Gram-negative rod [1,2,3]. It belongs to the class *Weeksellaceae,* within the family *Flavobacteriales* [1,2,3]. Although it commonly thrives in aquatic environments, *E. anophelis* has been isolated from both field-caught and laboratory-reared mosquitoes across diverse geographic regions [4,5]. Bacterial transmission between mosquitoes may occur vertically or horizontally [6,7,8,9]. *Elizabethkingia* significantly influenced host physiology including larval development, survival, and adult size in various vector mosquitoes [5,10,11]. *E. anophelis* has great potential to be utilized as a paratransgenesis agent [12]. For example, a recent study showed that *E. anophelis* exhibited broad-spectrum antiviral activity, inhibiting the replication of ZIKV, DENV, and CHIKV in vitro [13]. Furthermore, when introduced at a low bacterial dose, *E. anophelis* yielded a significant deleterious effect on *Plasmodium* parasite development, reducing the oocyst load [10]. It also demonstrated antibacterial properties, likely providing a competitive advantage in the mosquito midgut [9,14,15,16,17]. Therefore, *E. anophelis* imparts a “Swiss Army Knife” protective function against the viruses, parasites, and other pathogens that mosquitoes acquire and transmit [10,12,13].

Recent studies have shown that clinical human specimens including wound swabs, sputum, urine, body fluids, and blood frequently reveal the presence of *E. anophelis* [18,19]. Infections with *E. anophelis* pose a significant risk to individuals who are already ill, immunocompromised, or at age extremes [4,18,20]. Its causative diseases include neonatal meningitis, catheter-related bacteremia, and many others, leading to high mortality rates, ranging from 18% to 70% [6,20]. Moreover, a recent outbreak in the Upper Midwest region of the United States, specifically in Wisconsin, Illinois, and Michigan between 2015 and 2016, was attributed to *E. anophelis* [21]. In the Chicago metropolitan area, 14 people were sickened by *Elizabethkingia* in a ventilator-capable skilled nursing facility between 2021 and 2023 [22]. Several outbreaks have also been documented in Asia (Singapore, Taiwan, Hong Kong, and Mainland China), Europe, and Africa [11,20,21,23]. *Elizabethkingia* infections can apparently be acquired through both community and nosocomial settings, via exposure to contaminated surfaces of medical devices and equipment (such as hemodialysis and mechanical ventilation), water bodies and faucets, and the contaminated hands of healthcare workers [6]. Multiple transmission routes of *Elizabethkingia* to humans have been proposed [1,6]. An outbreak of *Elizabethkingia* infections has been linked to mosquitoes in the Central African Republic, while *E. anophelis* was further demonstrated to be transmitted from mosquitoes to mammalian hosts through mosquito bites [24,25]. However, the occurrence of several winter outbreaks may diminish the significance of this transmission route [21,22]. The above observations suggest that clinically important *E. anophelis* may have emerged from different lineages compared to mosquito-associated ones.

Several genomes of mosquito-associated *E. anophelis* strains have been sequenced, yet comprehensive genome analyses and systematic comparisons with clinically important strains have rarely been reported [11,26,27,28]. *E. anophelis* MSU001, a predominant bacterial member in the mosquito midgut, infected multiple mosquito species and was present in larval and adult life stages [9,17]. Therefore, it has great potential for the biocontrol of mosquito-borne disease. Moreover, it can be used as a model organism for studying microbe–mosquito interactions, due to its amenability for genetic manipulation [9,17]. In this study, we characterized a newly isolated strain and sequenced its genome to better understand its symbiotic traits. Furthermore, comparative genome analyses permitted investigation of its virulence factors and drug resistance, antecedent to applications as a paratransgenesis agent.

## 2. Materials and Methods

### 2.1. Culture

*E. anophelis* strain MSU001, the primary strain of focus in this study, was isolated from the dissected midguts of adult, female *Anopheles stephensi* Liston mosquitoes (Johns Hopkins strain) fed with 10% sucrose on the 7th day after adult emergence. It was held at a colony in an insectary at Michigan State University, using mosquito colonization methods and sterile techniques, as described elsewhere [9,17]. *E. anophelis* strain MSU001, *E. coli* JM109, and *Serratia marcescens* strain ano1 were grown in Luria–Bertani (LB) broth while shaking at 200 rpm at 30 °C [15]. Trypticase soy broth (TSB) medium was used for the culture of *Asaia* sp. W12 under the same conditions [15]. After MSU001 was cultured for 48 h, the spent broth was centrifuged at 4000 rpm for 15 min, filtered through a 2 µm filter, and heated at 80 °C for 10 min. To assess the effects of the spent medium on the growth of the tested bacteria including *E. coli*, *Serratia marcescens* ano1, and *Asaia* sp. W12, we added 100 µL of spent broth (prepared above) to 1.9 mL of bacterial suspension. After being cultured at 28 °C without shaking for 24 h, cell formation units (CFUs) were assayed by plating 100 µL of the above culture on their respective solid agars. For solid LB medium, Bacto agar (Difco, Detroit, MI, USA) was added at a final concentration of 20 g/liter and supplemented with erythromycin (Em) (100 µg/mL) for transposon selection. Previous studies showed that arginine is a critical amino acid that supports *E. anophelis* growth in M9 medium [9]. An arginine utilization-deficient mutant (strain SCH873) was obtained by transposon-directed (pHimarEm1) mutagenesis (Chen, unpublished). Strain SCH814 (as the wild-type control) had been previously created by conjugatively transferring a transposon carrying expression cassette *PompA + nluc* [9]. Both strains were used for metabolism experiments. For biochemical characterization of *E. anophelis* MSU001, we inoculated 150 μL of the bacterial suspension into a Biolog GEN III microplate and then incubated it at 30 °C. The color change was determined by following the manufacturer’s recommendation.

### 2.2. MALDI-ToF MS Analyses

*E. anophelis* strains were streaked onto separate sheep blood agar plates and incubated at a temperature of 35.5 °C. Individual colonies were chosen for identification through VITEK MS, a MALDI-TOF/MS system manufactured by BioMérieux in the USA. A small portion of a colony was applied to a target plate and then immediately covered with 1 μL of α-cyano-4-hydroxycinnamic acid matrix solution. After drying, the target plate was inserted into a VITEK mass spectrometer instrument. The resulting spectra were recorded in linear mode within a mass range of 2 to 20 kDa. The subsequent spectra were analyzed by comparing the characteristics of the obtained spectrum with the typical spectrum of each known species. The primary spectrum for MSU001 was compared to the VITEK MS MS-ID database (version 2.0) for identification.

### 2.3. Antibiotic Susceptibility Testing

A drug susceptibility panel was used to study the minimal inhibitory concentrations (MIC) of the selected isolates against antibiotics and antibacterial agents using a VITEK 2 system (BioMérieux, Durham, NC, USA). Then, 0.5 McFarland of bacterial inoculation was prepared, and the suspension was transferred into VITEK-2 AST-GN69 card. The antimicrobials included piperacillin/tazobactam, ticarcillin/clavulanic acid, trimethoprim/sulfamethoxazole, ampicillin/sulbactam, imipenem, ampicillin, piperacillin, meropenem, ceftazidime, aztreonam, cefepime, ceftriaxone, doripenem, ertapenem, cefazolin, amikacin, gentamicin, tobramycin, tetracycline, minocycline, tigecycline, levofloxacin, ciprofloxacin, and nitrofurantoin. The results were interpreted according to standards recommended by the Clinical and Laboratory Standards Institute (CLSI) for non-*Enterobacteriaceae*.

### 2.4. Genome Sequencing, Assembly, and Annotation

Next generation sequencing (NGS) libraries were prepared using an Illumina TruSeq Nano DNA Library Preparation Kit. Completed libraries were evaluated using a combination of Qubit dsDNA HS, Caliper LabChipGX HS DNA, and Kapa Illumina Library Quantification qPCR assays. Libraries were combined in a single pool for multiplexed sequencing, loaded on one standard MiSeq flow cell (v2), and sequencing was performed in a 2 × 250 bp paired-end format using a v2, 500 cycle reagent cartridge. NGS libraries were sequenced by Illumina Miseq paired-end sequencing technology at the Research Technology Support Facility (RTSF) at Michigan State University. The reads were assembled using CLC Genomics Workbench (version 10). Gene annotation was carried out using National Center for Biotechnology Information (NCBI) Prokaryotic Genome Automatic Annotation Pipeline (PGAAP 3.3) [29]. Initial prediction and annotation of coding sequences (CDS) and tRNA/rRNA gene prediction were carried out via Glimmer 3 through the Rapid Annotation using Subsystem Technology server (RAST) [30].

### 2.5. Bioinformatics

The selected genome sequences (Table 1) were downloaded from NCBI and annotated using Prokaryotic Genome Annotation Pipeline (PGAP) (version 6.5). The average GC contents, coding sequences, predicted genes, and genome size were predicted by PGAP. The functional categorization and classification of predicted CDS of MSU001 were performed on the RAST server-based SEED viewer [31]. The multi-drug resistance genes were predicted in the CARD database [31]. Prophages and clustered regularly interspaced short palindromic repeats (CRISPR) were predicted using CRISPRfinder [32]. For genomic similarity assessment, average nucleotide identity (ANI) and digital DNA-DNA hybridization (dDDH) values were computed using the web tools OrthoANIu and GGDC 2.0, respectively [33,34]. For quantification and classification of regulatory system proteins, the web tool P2RP was used [35]. The pan genome, core genome, and specific genes of MSU001 were analyzed by comparison with 16 representative *Elizabethkingia* genomes using EDGAR 3.2 [36]. Sizes of pan genomes and core genomes were estimated using the core/pan development feature [37].

Carbohydrate active enzyme families, including enzymes of glycan assembly (glycosyltransferases, GT) and deconstruction (glycoside hydrolases, GH, polysaccharide lyases, PL, carbohydrate esterases, CE), were semi-manually annotated using the Carbohydrate Active Enzyme (CAZy) database curation pipelines [38]. The metabolism pathways were predicted using antiSMASH (https://antismash.secondarymetabolites.org, accessed on 23 October 2023), RAST, gutSMASH (https://gutsmash.bioinformatics.nl, accessed on 23 October 2023), and previous metabolomics data. A phylogenetic tree of the 18 *Elizabethkingia* genomes was constructed based on the complete core genome. For all 2307 gene sets of the core genome, a multiple alignment was constructed using MUSCLE [37]. Subsequently, all alignments were concatenated and used as input for the neighbor joining method, as implemented in PHYLIP [39] and the approximate maximum likelihood method of Fasttree 2.1 [40]. The resulting phylogenies were basically identical. In total, 41,526 CDS were used, with 783,693 amino acid residues per genome, and 14,106,474 in total.

## 3. Results

### 3.1. Biochemical Characterization and Identification by MALDI-ToF/MS

**Figure 1:**
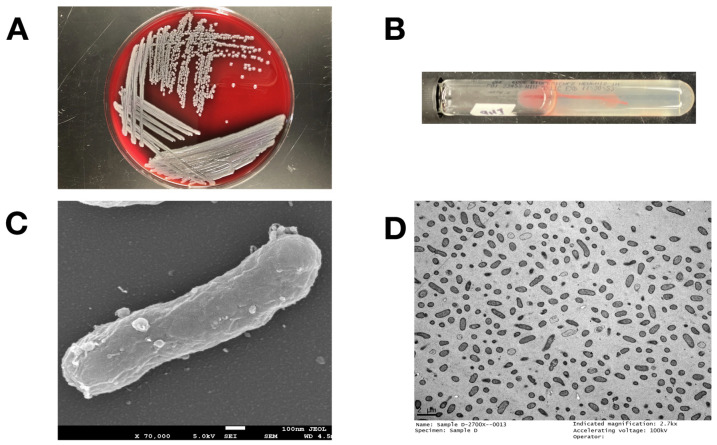
Growth features and microscopic observation of *E. anophelis* MSU001. (**A**) Hemolytic activity on sheep blood agar; (**B**) motility test; (**C**) scan electron microscopy; (**D**) demonstration of bacterial morphology by electron microscopy with negative stain.

*E. anophelis* MSU001 recovered from *A. stephensi* grew well in 5% sheep blood agar, without obvious hemolytic activity (Figure 1A) after 24 h incubation. It was nonmotile when cultured on motility test media (Figure 1B). It was oxidase positive and catalase positive. MSU001 cells were straight rods (Figure 1C,D) and had a diameter of 0.3 μm and length of 13.0 μm (Figure 1C). Carbon source (see Table S1), nitrogen source utilization, and osmotic tolerance were characterized by incubating cells in Biolog GEN III microplates at 37 °C overnight (Table S1). Our results showed that *E. anophelis* MSU001 tolerated up to 4% NaCl, but growth was inhibited at 8% NaCl. It metabolized several carbon sources, including the carbohydrates d-maltose, d-trehalose, d-cellobiose, d-gentibiose, d-sucrose, d-turanose, d-melibiose, d-glucose, d-mannose, d-fructose, d-fucose, d-mannitol, and d-glycerol. Moreover, it utilized d-serine, l-alanine, l-aspartic acid, l-glutamic acid, and l-histidine. The above observations indicated that *E. anophelis* MSU001 was capable of surviving in diverse environments.

The MALDI-TOF/MS system initially identified the strain as *Elizabethkingia meningosepticum* (Figure S1). However, analysis of the 16s rDNA sequence revealed a striking 99.93% similarity with *E. anophelis* Ag1 and *E. anophelis* R26, while only sharing an 80.37% similarity with *E. meningosepticum* strain NCTC10016 (ATCC 13253). This discrepancy can be attributed to the limitations of the default MALDI-ToF MS databases inaccurately classifying various members of the Flavobacteriaceae, particularly closely related strains within the *Chryseobacterium* and *Elizabethkingia* genera [41].

### 3.2. Genomic Features of E. anophelis MSU001

*E. anophelis* MSU001 had a genome size of 4.05 Mb and an average GC content of 35.4% (Table 1). The MSU001 genome encompassed 3857 coding sequences and 3753 genes. MSU001 possessed the second highest number of coding sequences (3857). The 17 selected *Elizabethkingia* genomes (comprising fourteen *E. anophelis*, two *E. meningoseptica*, and one *E. miricola*) exhibited similar general features (Table 1). These strains were isolated from diverse sources, such as mosquitoes, aquatic animals, plants, and humans in clinical settings. The genome sizes ranged from 3.59 to 4.42 Mb, with the GC content ranging between 35% and 36%. Among the mosquito-isolated *E. anophelis* strains (*n* = 6), the average genome size was 4.00 Mb. The genome size of *E. anophelis* MSU001 closely resembled those isolated from *A. gambiae* and *A. sinensis*, except for being slightly larger than *E. anophelis* As1. However, there was no statistically significant difference (*p* > 0.05, Student’s *t*-test) compared to the average genome size of 4.2 Mb (*n* = 5) observed in *E. anophelis* strains isolated from human clinical samples. The distribution of coding sequences among specific subsystems was predicted using SEED subsystems by RAST analysis (Supplemental Figure S2). This revealed 27 subsystems consisting of 87 categories. The major subsystems included “Amino acids and derivatives” (265 coding sequences), “Carbohydrates” (133 coding sequences), “Cofactors, vitamins, prosthetic groups, pigments” (131 coding sequences), and “Protein metabolism” (124 coding sequences). Notable subsystems also encompassed “Virulence, disease, and defense” (32 coding sequences) and several invasive genetic elements such as “Phages, prophages, transposable elements, plasmids” (24 coding sequences) (Figure S2). CRISPRs may alter the genome and modulate gene functions to serve as an adaptive immune system. MSU001 showed the presence of one CRISPR, while the other mosquito-associated isolates lacked any. Of the remaining *E. anophelis* isolates, CRISPRs were only seen in LDVH-AR107, 296-96, and SUE (each of which showed the presence of two CRISPRs). CRISPRs were otherwise only seen in *E. meningoseptica* strains (Table 1).

### 3.3. Gene Repertoire and Phylogenetic Interference of E. anophelis MSU001

**Figure 2:**
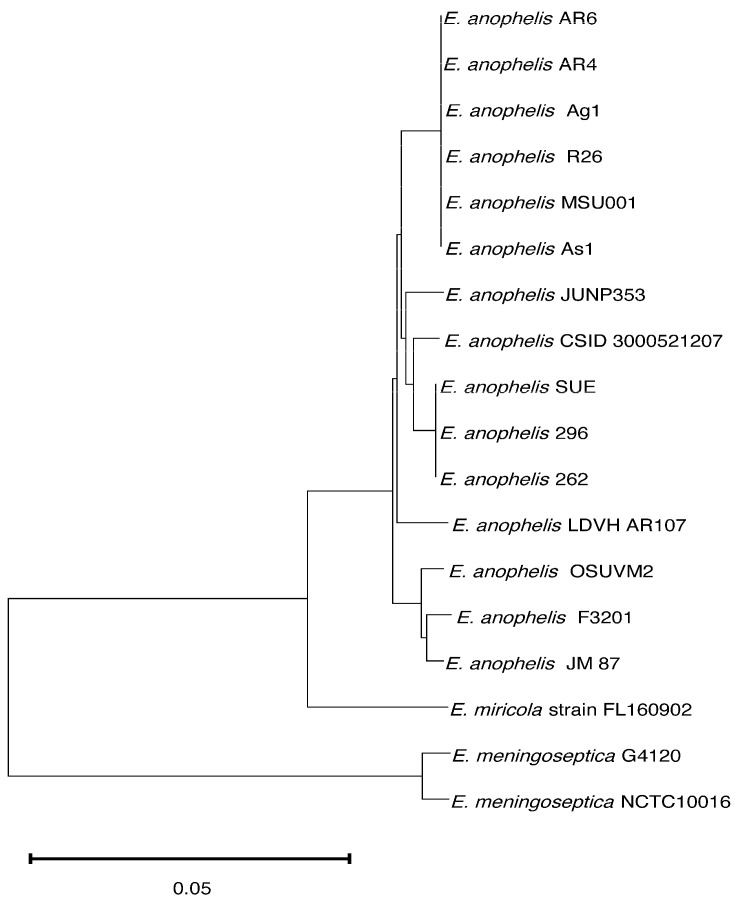
Phylogenetic placement of *E. anophelis* MSU001. The tree was constructed with 18 genomes with a core of 2307 genes per genome, 41,526 in total. The core had 783,693 amino acid residues/bp per genome, 14,106,474 in total. The horizontal bar represents 0.05 substitutions per site.

MSU001 showed a high ANI (>99%) with other strains of *E. anophelis* including R26 (type species), Ag1, AR4_6, AR6_8, and As1 (Table S2). The ANI value was greater than 97% for all other selected *E. anophelis* strains, indicating that MSU001 is indeed a strain of *E. anophelis*. However, ANI values were lower in comparison with *E. meningoseptica* (<81%) and *E. miricola* (<93%). Additionally, DDH values were calculated and were consistent with the analysis by ANI (Table S2). The phylogeny of selected *E. anophelis* strains is shown in Figure 2. *E. anophelis* MSU001 from *A. stephensi* was phylogenetically close to isolates from other mosquitoes (strain Ag1, R26, AR4-6, AR4-8 and As-1). The clinical strains were divided into three clusters and separated from the clade formed by mosquito isolates (Figure 2).

**Figure 3:**
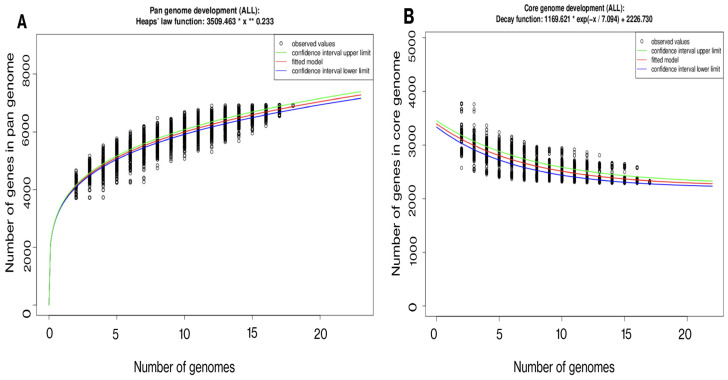
Pan and core genome evolution according to the number of selected *Elizabethkingia* genomes. (**A**) Number of genes (pan-genome) for a given number of sequentially added genomes. A pan development plot was generated for the following genomes: *E. anophelis* Ag1 (NZ_CP023402), *E. anophelis* R26 (NZ_CP023401), *E. anophelis* 2_62 (NZ_CP071551), *E. anophelis* 296_96 (NZ_CP046080), *E. anophelis* AR4_6 (NZ_CP023404), *E. anophelis* AR6_8 (NZ_CP023403), *E. anophelis* As1 (NZ_LFKT01000002), *E. anophelis* CSID_3000521207 (NZ_CP015067), *E. anophelis* F3201 (NZ_CP016375), *E. anophelis* JM_87 (NZ_CP016372), *E. anophelis* MSU001 (NZ_JAHDTL010000009), *E. anophelis* SUE (NZ_CP034247), *E. anophelis* LDVH-AR107 (NZ CP023403), *E. anophelis* JUNP 353 (NZ_ AP022313). (**B**) Number of shared genes (core genome) as a function of the number of genomes sequentially added. The genomes used for generating the core genome development plot were the same as listed in (**A**).

**Figure 4:**
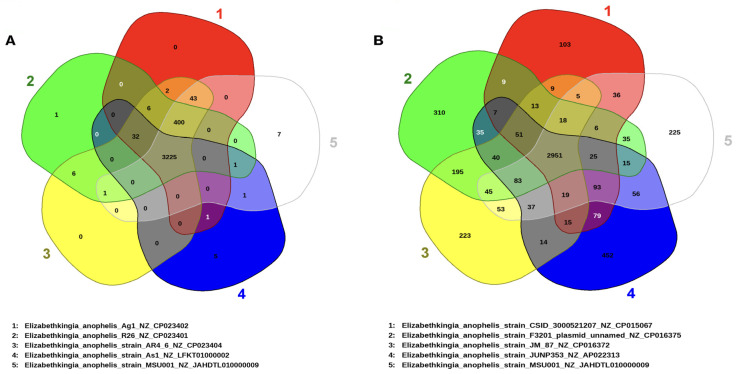
Venn diagram illustrating the distribution of shared and specific clusters of orthologous groups in the selected *Elizabethkingia* genomes. (**A**) Venn diagram of shared and unique genes in the selected mosquito-associated *Elizabethkiniga*. (**B**) Venn diagram of shared and unique genes in MSU001 and the clinically important *Elizabethkiniga*. The unique and shared genomes among the compared genomes were determined using the BLAST score ratio approach of EDGAR 3.2 with a cutoff of 30%.

The genomic elements encompassing the core and pan-genomes were organized and utilized to conduct an examination of the gene repertoire within selected genomes of *E. anophelis* (Figure 3A,B). Analysis of the core genome revealed a reduction in the shared gene count as more genomes were included in the analysis (Figure 3A). In general, *E. anophelis* exhibited characteristics of an open pan-genome, as evidenced by the appearance of new genes upon the addition of more sequenced genomes to the analysis (Figure 3B). Furthermore, the strain MSU001 (3678) shared 3668, 3627, 3669, and 3669 genes in common with the mosquito isolates Ag1, R26, AR4, and AR6, respectively (Figure 4A). These commonly shared genes accounted for approximately 99.7%, 98.6%, 99.8%, and 99.8% of the encoding genes of MSU001, respectively. It shared 3225 common genes with As1, which is ~87.7% of the common encoding genes of MSU001, due to the small genome size of As1. However, MSU001 shared far fewer genes with clinical *E. anophelis* strains (Figure 4B) including CSID_3000521207 (3153), JUNP 353 (3257), F3201 (3165), 296-96 (3266), and SUE (3264). These accounted for less than 85.7%, 88.6%, 86.1%, 88.8%, and 88.7% of the MSU001 encoding genes, respectively. Even fewer genes were shared between isolates found in other hosts such as LDVH-AR107 (3193), OSUVM 2 (3117), and JM-87 (3195). These accounted for less than 84.7%, 84.7%, and 86.9% of MSU001 encoding genes (Figure S3), respectively.

### 3.4. Metabolites Involved in Symbiosis

Several important metabolites such as sphingolipids (SLs) and inositol were detected in the extracts from the midguts of mosquitoes which were fed with both sugar and blood meals in a previous study [42]. Genes involved in the biosynthesis of SLs and inositol were detected in *E. anophelis* genomes, highlighting that *E. anophelis* may contribute to the above process. Although SLs are not commonly found as components of bacterial membranes, they have been uniquely identified in certain groups of microbes such as *Bacteroides* and *Sphingomonads* [43]. Interestingly, the putative sphingolipid synthesis genes were identified in all selected *Elizabethkingia* genomes, suggesting their potential involvement in symbiotic relationships, affecting cytotoxicity, colonization of the host, biofilm formation, and modulation of host inflammation [44]. Furthermore, inositol, an important nutritional and signaling factor, was found to be involved in metabolic pathways [45]. These pathways may participate in regulating the stress response, such as cold tolerance, in the hosts.

**Figure 5:**
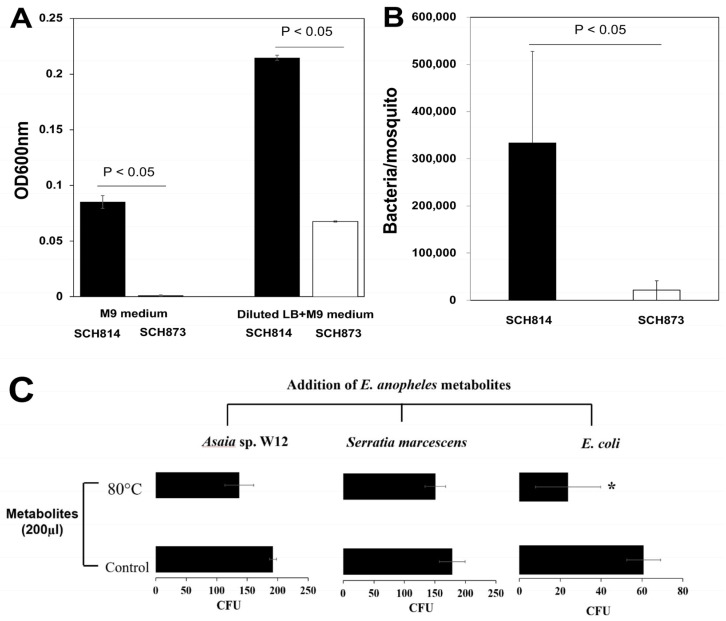
Inhibitory effects of *Elizabethkingia* metabolites on selected bacteria. * Statistically significant difference (*p* < 0.05). (**A**) Growth comparison between wild type strain for arginine utilization (SCH814) and arginine metabolism mutant (SCH873) in the M9 medium and M9 medium supplemented with 20-fold diluted LB medium. (**B**) Comparison between growth of SCH814 and SCH873 in mosquitoes. (**C**) The effects of spent media on the growth of *Asaia* sp. W12, *Serratia marcescens* and *E. coli.* The spent broth from *E. anophelis* MSU001 (48-h incubation) was added *E. coli*, *Serratia marcescens* ano1 and *Asaia* sp. W12, statically cultured at 28 °C for 24 h and plated on their respective solid agar media for CFU calculation.

The growth of SCH873 in M9 medium was impaired, compared to the WT (SCH814) (Figure 5A, left panel). When a 20-diluted LB broth was added into M9 medium, the growth of SCH873 was promoted, while the cell density was much lower than that in SCH814 (Figure 5A, right panel). At 7 days post-infection in adult mosquitos, the cell density of WT *Elizabethkingia* cells was around 15.8-fold higher than that of arginine utilization mutants in *A. stephensi*, indicating that *Elizabethkingia* cells might need to interact with either mosquito host or other microbes to obtain arginine for growth (Figure 5B). To assess the effects of *E. anophelis* metabolites on the growth of other common mosquito gut symbionts (*Asaia* sp. W12 and *Serratia marcescens*), the number of colonies that grew from cultures with added metabolites was compared to control groups (Figure 5). In cultures of *E. coli* (a representative for non-symbionts), the metabolites significantly hindered colony formation, resulting in less than half the number of viable colonies compared to the control group and indicating a reduction in growth by approximately 58%. The growth inhibition of *Asaia* sp. W12 and *Serratia marcescens* with metabolites was less pronounced, with approximately 26% and 17% reductions in growth (Figure 5C), respectively. These findings suggest that *E. anophelis* metabolites have inhibitory effects on the growth of common mosquito gut symbionts, highlighting the potential role of *E. anophelis* in modulating the microbial community within the mosquito gut.

### 3.5. Regulatory System Proteins

The genome of *E. anophelis* MSU001 possessed genes encoding 51 two-component system proteins, 188 transcription factor proteins, and 13 other DNA-binding proteins, resulting in a total count of 252 regulatory proteins (Table 2). This count was the highest among the mosquito-associated *E. anophelis* isolates, except for As1, which displayed reduced protein counts in all categories, totaling 215 proteins (Table 2). The other mosquito-associated isolates shared similar counts of two-component system proteins and transcription factor proteins. The main variation among these isolates was observed in the number of DNA-binding proteins, with Ag1, AR4-6, and AR6-8 lacking only one fewer ODP (another DNA-binding protein), and R26 lacking two (Table 2).

### 3.6. Carbohydrate Active Enzymes

A total of 124 CAZyme-encoding genes were predicted in *E. anophelis* MSU001, consisting of approximately 3% of the bacterial genome (Tables S3 and S4). Notably, CBM12 (carbohydrate-binding module family 12) and AA10 (auxiliary activity family 10, lytic polysaccharide monooxygenases) were exclusive to mosquito-associated *E. anophelis* strains, highlighting their importance in establishing a symbiotic relationship with insects. The overall predicted CAZyme repertoires in mosquito-associated *E. anophelis* were comparable, featuring 61 glycoside hydrolases (GHs). In contrast, *E. anophelis* As1 exhibited a slightly lower count of 56 GHs (Table S3). This collective decrease in GHs among mosquito isolates, ranging from 61 to 67, contrasted with clinical species, suggesting a distinct evolutionary route. Compared to the clinically important strains, decreased copy numbers of GH3, GH29, and GT4 were detected in insect-associated *Elizabethkingia* strains (Table S3), showing that while these specific CAZyme genes may be involved in pathogenesis in humans, they may not be relevant for insect symbiosis. Both *E. anophelis* and *E. miricola* species harbored single copies of GH1 (β-glycosidase), which is absent in *E. meningoseptica*. Conversely, GH30, present in *E. meningoseptica*, was only detected in selected clinical *E. anophelis* strains and was absent in *E. miricola*. Additionally, *E. anophelis* lacked GH33 (sialidase), a characteristic found in *E. meningoseptica* and some *E. miricola* strains. Genes encoding GH5 (subfamily 46) and CBM6 (β-glucan binding), consistently observed in *E. anophelis*, were not found in *E. meningoseptica*.

### 3.7. Pathogenesis Potential Revealed by Virulence Factors and MDR Analysis

Using the VFDB protein Set B database, a comparative analysis of selected *Elizabethkingia* isolates was conducted to identify homologs of virulence factors (VFs) (Table 3). Ten VFs of interest were discovered, namely C8J 1080, DnaK, EF-Tu, eno, htpB, katG, mps1-1, mps1-2, pgIC, and RmIA. These VFs play diverse roles in cellular functions such as mitotic regulation, capsule formation, stress response (involving heat shock proteins, catalase, and hydratase), ion transport proteins, secretion systems, and defense or invasion mechanisms during pathogenesis. Among the selected VFs, genes encoding DnaK, EF-Tu, mps1-1, mps1-2, and RmIA were present in all *E. anophelis* isolates. Eno and htpB were found in all mosquito-associated isolates, while their presence in clinically isolated human samples varied. PgIC was observed in all mosquito-associated isolates but was completely absent in human *Elizabethkingia* strains. Both mosquito- and human-associated *E. anophelis* strains shared the presence of C8J 1080 and katG, which were not identified in other animal-associated strains (Table 3).

The antimicrobial resistance profile of *E. anophelis* was determined using the broth microdilution method. The strain exhibited resistance to 13 out of the 16 tested antibiotics, including aminoglycosides, tetracycline, nitrofuran, and all β-lactam antibiotics, such as cephalosporins, monobactams, and extended-spectrum penams/β-lactamase inhibitors. However, it showed susceptibility to trimethoprim/sulfamethoxazole (sulfonamide) and ciprofloxacin (quinolone), and intermediate susceptibility to tigecycline (Table 4). In addition, the prediction of antibiotic resistance genes in *E. anophelis* MSU001 revealed its multidrug resistance traits (Table S4). Notably, *Elizabethkingia* species are known for their high resistance to β-lactam drugs, due to the production of β-lactamases (Table S4), which hydrolyze these antibiotics. In the case of MSU001, it carried at least five different β-lactamase genes (BlaB, CME-1, GOB-9, IND-7, and TLA-1) that may confer broad resistance to penams, cephalosporins, and carbapenems. It is interesting that the presence of IND-7, which encodes for a class B carbapenem-hydrolyzing β-lactamase, was unique to the MSU001 strain. Mosquito-associated *E. anophelis* strains carried GOB-9 (encoding a class B β-lactamase) and TLA-1, which were only found in a few clinical *Elizabethkingia* isolates. Furthermore, it is noteworthy that GOB-9 was absent in *E. miricola* and *E. meningoseptica*. Genes encoding BlaB (inducible class C cephalosporinase) and CME-1 (class A β-lactamase) were present in most selected *Elizabethkingia* species (Table S4). However, mosquito-associated *E. anophelis* lacked several β-lactamase genes found in other selected *Elizabethkingia* strains, indicating unique evolutionary routes for these mosquito-associated strains.

## 4. Discussion

Studies have shown that a substantial portion of the colonizing bacteria found within adult mosquito hosts are acquired in aquatic habitats during larval life stages [9,16,17]. *Elizabethkingia* species are common mosquito symbionts dispersed in natural water bodies (dams, wetlands, and rivers), but do not normally predominate in these environments (composing 6.25 × 10^−6^ to 8.21 × 10^−6^ of the total bacterial community) [46,47]. However, *Elizabethkingia* species populate mosquito midguts and can spread to other organs and tissues, including the salivary glands, reproductive organs (ovary or testicles), crop, and alimentary canal of mosquitoes at various development stages [47]. The complex interactions between arthropod hosts and their associated microbes warrant a holistic analysis of these communities and the environments that foster them [47]. Bacteria need to overcome digestion, microbial competition, and a multitude of other stress factors (e.g., iron and oxidative stress, larval metamorphosis, temperature, pH) associated with mosquito physiology [9,17]. The ability to thrive in dynamic environments within a host emphasizes the importance of bacterial adaptability and likely highlights a deeper symbiotic relationship underlying microbial persistence [47]. By conducting an analysis of the genomic and molecular mechanisms behind *Elizabethkingia* colonization, we hoped to enhance our understanding of microbe–host interactions.

Correctly identifying *Elizabethkingia* species has proven to be a challenge with varying success, further complicated by prior nomenclature changes and various method limitations [41]. Current classification of *Flavobacteriaceae* members relies heavily on MALDI-ToF mass spectrometry, but despite its wide utility in bacterial identification, it struggles to accurately classify members from *Chryseobacterium* and *Elizabethkingia* genera [19,41,46]. Furthermore, standard databases are limited to only a few *Elizabethkingia* isolates, often falsely defaulting to *E. meningoseptica* or *E. miricola* [41]. This was evidenced by our own study, as well as others, where MALDI-ToF frequently misidentified *E. anophelis* as *E. meningosepticum* [41,46,48]. The use of 16S rRNA sequences has been shown to be limited in its taxonomic utility as well [48]. The fact that misidentification via conventional methodologies is so prevalent in the literature may indicate *E. anophelis* is an underrepresented pathogen responsible for more disease in humans than previously attributed [46]. These limitations highlight the need for updating standard MALDI-ToF databases, as well as for thorough, enhanced identification methodologies that utilize a combination of widely adopted bacterial identification methods like 16s rDNA sequencing in conjunction with biochemical testing [41,46,48]. Moreover, whole genomic sequence analysis and average nucleotide identity as a complementary method may be used to correctly identify *E. anophelis* [46,49].

Genome size and GC content were similar among most *E*. *anophelis* strains. MSU001 exhibited characteristics of an open pan-genome, likely relating to its diverse habitats, spanning both aquatic and terrestrial environments, as well as the many different human, animal, and plant hosts that it may colonize [46]. However, the core genome analysis demonstrated that strains from mosquitoes shared more conserved genes than those from clinical specimens. Furthermore, the phylogenetic placement of mosquito-associated *E. anophelis* species formed different clades from clinical isolates. They were also distinct from *E. meningoseptica* and *E. miricola* clades. Collectively, these results indicate that *E. anophelis* MSU001 and other mosquito isolates likely evolved in different routes to adapt to mosquito hosts compared to clinical strains.

Another notable finding was the presence of *Elizabethkingia* genes involved in sphingolipid biosynthesis. Sphingolipids are a ubiquitous component in eukaryotic cell membranes that have been shown to play critical roles in cell signal transduction, regulation of apoptosis, adhesion and uptake, and inflammation in the host [50]. Several pathogens can actively synthesize or hydrolyze these molecules to hijack host cell responses and orchestrate favorable immune responses [50]. Furthermore, certain sphingolipids like sphingosine have also been shown to possess a possible antibacterial effect [50]. Bacteria employ diverse mechanisms to facilitate host interactions and survival in their environments. The production of various secondary metabolites by *Elizabethkingia* likely conferred advantages over other members of the microbial community, allowing it to disturb the bacterial consortium and outcompete or even inhibit its competitors [50].

Chitin is one of the most abundant polysaccharides, forming important structures in the insect exoskeleton and gut linings [51]. Due to the vital role of chitin in development and defense against pathogen invasion, insects need to frequently reshape its structure and components [51]. Microbial symbionts may be involved in chitin degradation and its synthesis [52]. In this study, we observed that the modules of CBM12 associated with chitinase and AA10 were uniquely found in mosquito-associated *E. anophelis* (except As1). These CAZymes possibly contribute to the binding and lysing of chitin [52]. For example, upon a mosquito’s bite, the ingested blood meal triggers the midgut epithelium to release various factors including chitin microfibrils (3–13%) and protein complexes, which form a peritrophic matrix (PM) [53]. The PM effectively creates a barrier between the blood bolus and the midgut epithelial cells, serving as a protective shield against abrasive particles and microbial infections [53]. After the red blood cells have been thoroughly digested, the PM needs to be dismantled to release the nutrients. Microbial chitinase secreted by gut microbiota may facilitate this process [52,53,54]. Moreover, microbial chitinases may contribute to the reshaping of chitin components during mosquito molting, supported by the presence of *E. anophelis* in various mosquito body sites [51,52]. The majority of predicted CAZymes in *Elizabethkingia* species appear to be involved in utilizing simple sugars rather than degrading complex plant polysaccharides, which is consistent with their living niches (e.g., within mosquitoes or humans) [46,47,48]. Our results also indicated that pathogenic *E. anophelis* possibly requires additional copies of GH3, GH29, and GT4 to participate in pathogenesis. Furthermore, *E. anophelis* and *E. miricola* have different sets of CAZymes involved in sugar metabolism. Therefore, future characterization of their physiological functions is warranted.

Despite their different sources, *Elizabethkingia* bacteria exhibited comparable numbers of response regulators, phosphotransferase proteins, histidine kinases, one-component systems, transcriptional regulators, sigma factors, and other DNA-binding proteins (Table 2). These regulatory proteins play critical roles in maintaining bacterial metabolism and function, explaining their consistent presence across *Elizabethkingia* species (Table 2). The numbers of regulatory protein genes between mosquito-associated and clinical *E. anophelis* genomes varied and were not statistically different. The retainment of similar complicated regulatory systems may indicate an adaptability of this organism to diverse host environments [46]. *E. anophelis* living in the adult female mosquito midgut may experience similar stress conditions to those where bacteria invade the bloodstream of mammalian hosts [9,16,17]. For example, mosquito-associated bacteria are exposed to iron-depleting conditions and relatively lower temperatures prior to blood meals [13,17]; conversely, they encounter iron-rich environments during and after blood meals [13]. Similar processes may occur prior to entry into the bloodstream or after the lysis of the erythrocytes during a bacteremia event [25,28]. Furthermore, the evasion of immune cells and resistance to temperature variations during the above processes are expected to be similar [55].

The emerging pathogenicity of *Elizabethkingia* is likely attributed to its large genome, ecological and metabolic plasticity, a multitude of virulence factor genes present in its genetic repertoire, and broad antibiotic resistance [46,48]. Among the diverse virulence factors, we discovered that PgIC was only present in mosquito-associated isolates. PglC plays a vital role in the N-linked protein glycosylation pathway in *Campylobacter jejuni* [56]. This pathway primes proteins for nucleophilic attack by the polyprenol acceptor within the cellular membranes, which may play important roles in epithelial cell adherence, invasion, and colonization of the host during the infection course [56,57]. Antimicrobial susceptibility patterns vary across strains and in the case of clinical isolates, provide an additional layer of difficulty in the selection of appropriate therapeutics [23,58,59]. While β-lactamase synthesis remains the most employed defense among Gram-negative bacteria to withstand antibiotics, other resistance mechanisms include the alteration of target drug sites and the implementation of efflux pumps to eliminate the drug from the cell [59]. The presence of specific β-lactamase genes varies across different host-associated strains, suggesting that these genes confer certain advantages within *Elizabethkingia* and their respective evolutionary routes [20,23]. Those virulence factors that aid in transmission promote adhesion, motility, and biofilm formation, while other factors mediate host interactions and allow for extended persistence within hostile environments [58,60]. Further research into variations in genomic features between mosquito-associated and clinically significant strains of *Elizabethkingia* is warranted.

## Figures and Tables

**Table 1:** Genomic features in selected *Elizabethkingia* species.

Strain	Original Region *^a^	Isolation Source *^b^	Size (Mb)	GC%	CDS	Gene	CRISPR Count
*E. anophelis*							
As1	USA	*A. gambiae*	3.59	35.5	3237	3315	0
Ag1	USA	*A. gambiae*	4.09	35.5	3686	3788	0
R26	Sweden	*A. gambiae*	4.06	35.5	3635	3741	0
AR4-6	China	*A. sinensis*	4.09	35.5	3678	3785	0
AR6-8	China	*A. sinensis*	4.09	35.5	3678	3785	0
MSU001	USA	*A. stephensi*	4.05	35.4	3857	3753	1
LDVH-AR107	France	*C. carpio*	3.99	35.7	3555	3667	2
OSUVM 2	USA	*E. caballus*	4.1	35.4	3644	3754	0
CSID_3000521207	USA	*H. sapiens*	3.85	35.7	3412	3513	0
JUNP 353	Nepal	*H. sapiens*	4.32	35.8	3897	4049	0
F3201	Kuwait	*H. sapiens*	4.28	35.46	3797	3927	0
296-96	Taiwan	*H. sapiens*	4.2	35.8	3779	3898	2
SUE	Taiwan	*H sapiens*	4.2	35.8	3771	3891	2
JM-87	USA	*Z. mays*	4.18	35.5	3695	3837	0
*E. meningoseptica*							
NCTC10016	UK	*H. sapiens*	3.87	36.5	3397	3480	1
G4120	France	*H. sapiens*	4	36.4	3519	3628	1
*E. miricola*							
FL160902	China	Frog	4.25	35.7	3760	3892	0

*^a,b^ The information about specimen and sources used for these selected isolates was obtained from BioSample (https://www.ncbi.nlm.nih.gov/biosample).

**Table 2:** Predicted regulatory proteins in the selected *Elizabethkingia* species *.

Elizabethkingia	Predicted Regulatory Proteins							
	TOC			TF				ODP
	RR	PP	HK	OCS	RR	TR	SF	
*E. anophelis*								
Ag1	26	9	16	31	23	118	16	12
As1	23	8	14	22	20	103	15	10
R26	26	9	16	31	23	118	16	11
AR4-6	26	9	16	31	23	118	16	12
AR6-8	26	9	16	31	23	118	16	12
MSU001	26	9	16	31	23	118	16	13
LDVH-AR107	26	8	17	26	23	119	17	12
OSUVM 2	29	9	21	32	26	128	18	8
CSID_3000521207	27	8	17	27	23	113	16	10
JUNP 353	27	8	18	30	23	117	17	11
F3201	18	9	20	30	25	133	16	12
296-96	26	7	19	29	22	119	18	10
SUE	27	7	18	29	23	118	18	11
JM-87	30	9	21	28	27	124	18	9
*E. meningoseptica*								
NCTC10016	19	29	10	27	25	117	15	6
G4120	28	10	18	16	15	121	16	6
*E. miricola*								
FL160902	35	11	25	31	31	131	20	10

* The regulatory proteins were predicted by the web tool P2RP [35]. TOC, two-component systems; TF, transcription factors; ODP, other DNA-binding proteins; RR, response regulators; PP, phosphotransferase proteins; HK, histidine kinases; OCS, one-component systems; TR, transcriptional regulators; SF, sigma factors. The numbers in this table are the gene copies encoding the regulatory proteins.

**Table 3:** Selected virulence factors in *Elizabethkingia* species *.

	C8J 1080	DnaK	EF-Tu	eno	htpB	katG	mps1-1	mps1-2	pglC	RmlA
*E. anophelis*										
As1	+	+	+	+	+	+	+	+	+	+
Ag1	+	+	+	+	+	+	+	+	+	+
R26	+	+	+	+	+	+	+	+	+	+
AR4-6	+	+	+	+	+	+	+	+	+	+
R6-8	+	+	+	+	+	+	+	+	+	+
MSU001	+	+	+	+	+	+	+	+	+	+
LDVH-AR107	+	+	+	-	-	-	+	+	-	+
OSUVM 2	-	+	+	+	+	+	+	+	-	+
CSID_3000521207	+	+	+	+	+	+	+	+	-	+
JUNP 353	+	+	+	-	-	+	+	+	-	+
F3201	+	+	+	+	+	+	+	+	-	+
296-96	+	+	+	-	-	+	+	+	-	+
SUE	+	+	+	-	-	+	+	+	-	+
JM-87	+	+	+	-	-	+	+	+	-	+
*E. meningoseptica*										
NCTC10016	-	+	+	-	-	+	-	-	-	+
G4120	-	+	+	-	-	-	-	-	-	-
*E. miricola*										
FL160902	-	-	+	-	-	-	+	+	-	-

* + indicates the presence; - indicates the absence.

**Table 4:** Antimicrobial susceptibility test.

Antibiotic Class	Antimicrobial	MIC (µg/mL) *	SIR
Aminoglycosides			
	Amikacin	≥64	R
	Gentamicin	≥16	R
β-lactams and β-lactamase inhibitors			
	Meropenem	≥16	R
	Cefazolin	≥64	R
	Cefotaxime	≥32	R
	Tobramycin	≥16	R
	Aztreonam	≥64	R
	Ampicillin	≥32	R
	Ampicillin/Sulbactam	≥32	R
	Piperacillin	≥64	R
	Ceftriaxone	≥64	R
	Piperacillin/Tazobactam	≥128	R
Sulfonamide	Trimethoprim/Sulfamethoxazole	40	S
Quinolone	Ciprofloxacin	0.5	S
Tetracycline	Tigecycline	4	I
Nitrofuran	Nitrofurantoin	128	R

* Minimum inhibitory concentration (μg/mL) was determined by the VITEK. S, I, and R stand for sensitive (S), intermediately sensitive (I), and resistant (R), respectively. The results were interpreted using the Clinical and Laboratory Standards Institute (CLSI) for non-*Enterobacteriaceae*.

## Data Availability

Data from these whole-genome shotgun projects have been deposited at DDBJ/ENA/GenBank under accession number GCA_024357565.1. The BioProject designation for this project is PRJNA731841, and the BioSample accession number is SAMN19296199.

## Supplementary Materials

The following supporting information can be downloaded at https://www.mdpi.com/article/10.3390/microorganisms12061079/s1. Table S1: Biolog tests of *E. anophelis* MSU001; Table S2: Average nucleotide identity values (up, black font) and Digital DNA-DNA Hybridization values (low, red font) amongst different *Elizabethkingia* species; Table S3: CAZyase analyses in the selected *Elizabethkinigia*; Table S4: Resistome analysis of *Elizabethkingia* spp.; Figure S1: mass spectrum; Figure S2: 27 subsystems consisting of 87 categories; Figure S3: Agininine prediction by gutSMASH.
