# Supplementary material for: Elizabethkingia anophelis MSU001 Isolated from Anopheles stephensi: Molecular Characterization and Comparative Genome Analysis
Source: Microorganisms. 2024 May 27;12(6):1079. doi: 10.3390/microorganisms12061079 (PMC11206156; doi:10.3390/microorganisms12061079)
Supplement: Supplementary file 1 [file microorganisms-12-01079-s001.zip › Figure S1 Mass spectrum.pptx]

## Slide 1
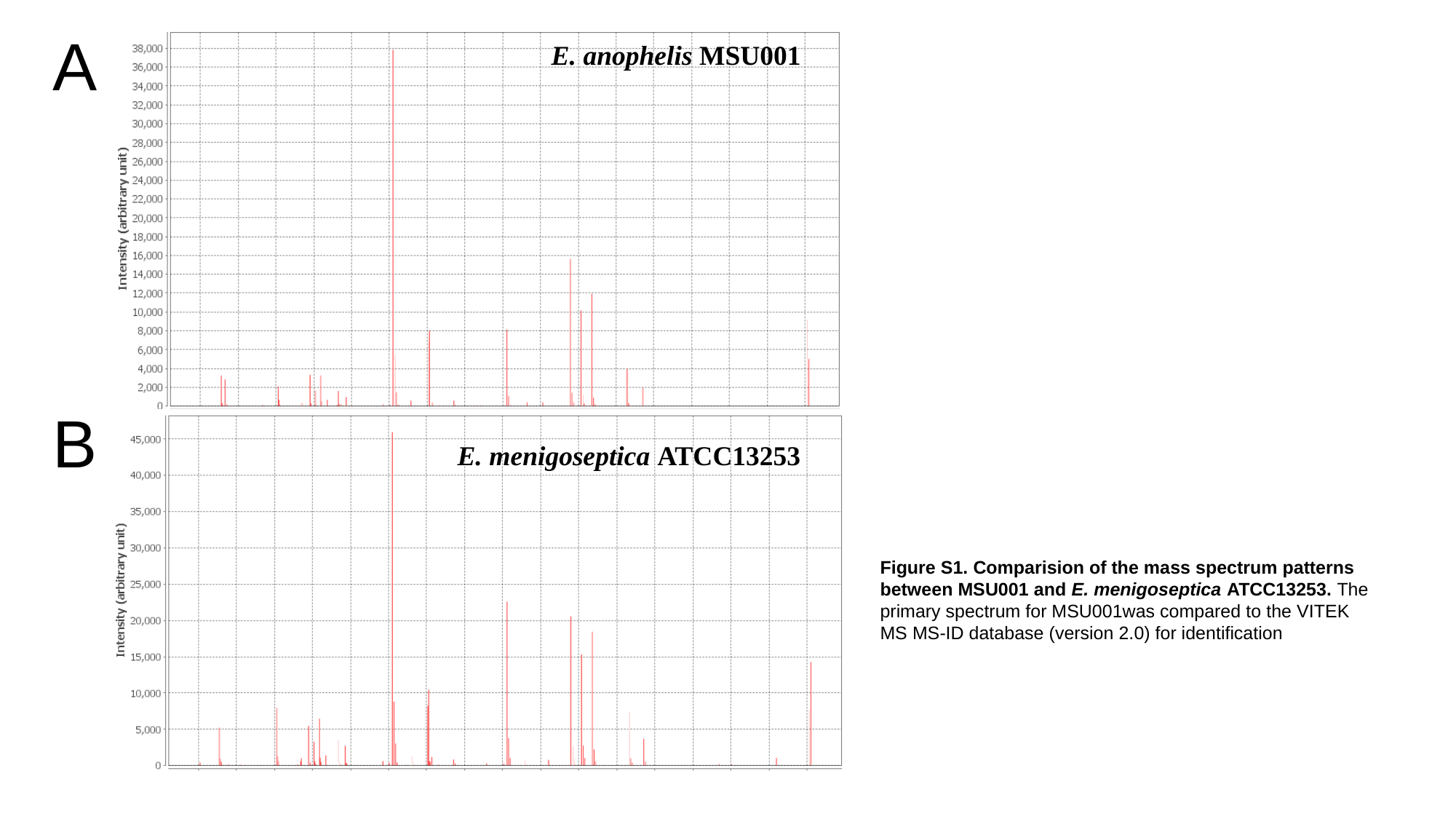

A
E. anophelis MSU001
B
E. menigoseptica ATCC13253
Figure S1. Comparision of the mass spectrum patterns between MSU001 and E. menigoseptica ATCC13253. The primary spectrum for MSU001was compared to the VITEK MS MS-ID database (version 2.0) for identification
