# Supplementary material for: Elizabethkingia anophelis MSU001 Isolated from Anopheles stephensi: Molecular Characterization and Comparative Genome Analysis
Source: Microorganisms. 2024 May 27;12(6):1079. doi: 10.3390/microorganisms12061079 (PMC11206156; doi:10.3390/microorganisms12061079)
Supplement: Supplementary file 1 [file microorganisms-12-01079-s001.zip › Figure S2 Supplemental materials 27 subsystems consisting of 87 categories.pdf]

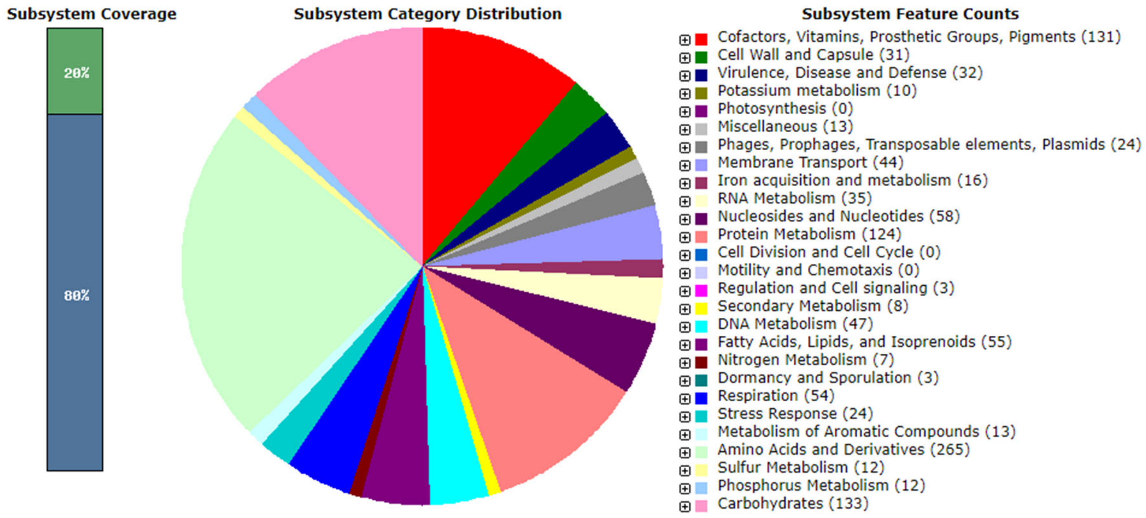

Figure S2. Subsystem category distribution of *E. anophelis* MSU001 using SEED subsystems by RAST analysis. The pie chart represents relative abundance of each subsystem category and numbers depict subsystem feature counts.
