# Supplementary material for: Elizabethkingia anophelis MSU001 Isolated from Anopheles stephensi: Molecular Characterization and Comparative Genome Analysis
Source: Microorganisms. 2024 May 27;12(6):1079. doi: 10.3390/microorganisms12061079 (PMC11206156; doi:10.3390/microorganisms12061079)
Supplement: Supplementary file 1 [file microorganisms-12-01079-s001.zip › Figure S3.pptx]

## Slide 1
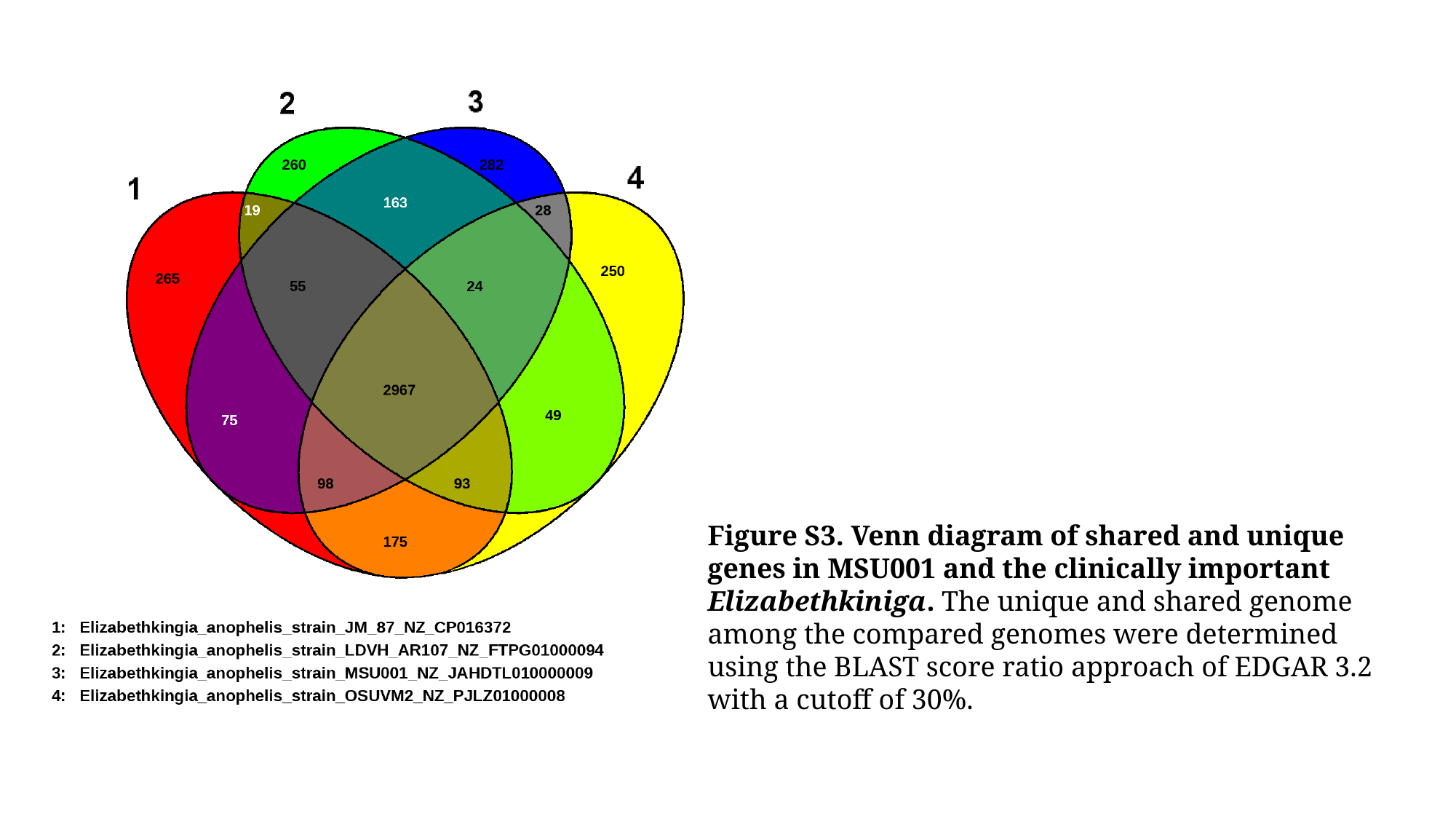

Figure S3. Venn diagram of shared and unique genes in MSU001 and the clinically important Elizabethkiniga. The unique and shared genome among the compared genomes were determined using the BLAST score ratio approach of EDGAR 3.2 with a cutoff of 30%.
