# Supplementary material for: Elizabethkingia anophelis MSU001 Isolated from Anopheles stephensi: Molecular Characterization and Comparative Genome Analysis
Source: Microorganisms. 2024 May 27;12(6):1079. doi: 10.3390/microorganisms12061079 (PMC11206156; doi:10.3390/microorganisms12061079)
Supplement: Supplementary file 1 [file microorganisms-12-01079-s001.zip › Table S1 .pdf]

Table S1 Biolog tests of *E. anophelis* MSU001

	1	2	3	4	5	6	7	8	9	10	11	12
A	**Negative Control**<br>No Growth	**Dextrin**<br>+++	**D-Maltose**<br>++	**D-Trehalose**<br>++	**D-Cellobiose**<br>+	**Gentiobiose**<br>++	**Sucrose**<br>++	**D-Turanose**<br>++	**Stachyose**<br>++	**Positive Control**<br>+++	**pH 6**<br>+++	**pH 5**<br>No Growth
B	**D-Rafinose**<br>No Growth	**α -D-Lactose**<br>++	**D-Melibiose**<br>++	**β-Methyl-D-Glucoside**<br>++	**D-Salicin**<br>++	**N-Acetyl-D-Glucosamine**<br>++	**N-Acetyl-β-D-Mannosamine**<br>++	**N-Acetyl-D-Galactosamine**<br>++	**N-Acetyl-Neuraminic Acid**<br>++	**1% NaCl**<br>+++	**4% NaCl**<br>+	**8% NaCl**<br>+
C	**D-Glucose**<br>++	**D-Mannose**<br>++	**D-Fructose**<br>++	**D-Galactose**<br>++	**3-Methyl Glucose**<br>++	**D-Fucose**<br>++	**L-Fucose**<br>++	**L-Rhamnose**<br>++	**Inosine**<br>++	**1% Sodium Lactate**<br>+++	**Fusidic Acid**<br>++	**D-Serine**<br>+++
D	**D-Sorbitol**<br>+	**D-Mannitol**<br>++	**D-Arabitol**<br>++	**Myo-Inositol**<br>++	**Glycerol**<br>++	**D-Glucose-6-PO_4**<br>+	**D-Fructose-6-PO_4**<br>++	**D-Aspartic Acid**<br>+	**D-Serine**<br>++	**Troleandomycin**<br>++	**Rifamycin SV**<br>++	**Minocycline**<br>++
E	**Gelatin**<br>+++	**Glycyl-L-Proline**<br>++	**L-Alanine**<br>++	**L-Arginine**<br>++	**L-Aspartic Acid**<br>++	**L-Glutamic Acid**<br>++	**L-Histidine**<br>++	**L-Pyroglutamic Acid**<br>++	**L-Serine**<br>++	**Lincomycin**<br>++	**Guanidine HCl**<br>++	**Niaproof 4**<br>No Growth
F	**Pectin**<br>+	**D-Galacturonic Acid**<br>++	**L-Galactonic Acid Lactone**<br>+	**D-Gluconic Acid**<br>++	**D-Glucuronic Acid**<br>++	**Glucuronamide**<br>++	**Mucic Acid**<br>+	**Quinic Acid**<br>++	**D-Saccharic Acid**<br>++	**Vancomycin**<br>++	**Tetrazolium Violet**<br>+++++	**Tetrazolium Blue**<br>++++++
G	**p-Hydroxy-Phenylacetic Acid**<br>No Growth	**Methyl Pyruvate**<br>+	**D-Lactic Acid Methyl Ester**<br>+	**L-Lactic Acid**<br>++	**Citric Acid**<br>++	**α-Keto-Glutaric Acid**<br>++	**D-Malic Acid**<br>+	**L-Malic Acid**<br>+	**N-Bromo-Succinic Acid**<br>No Growth	**Nalidixic Acid**<br>++	**Lithium Chloride**<br>++	**Potassium Tellurite**<br>+++
H	**Tween 40**<br>++	**γ -Amino-Butyric Acid**<br>No Growth	**α-Hydroxy-Butyric Acid**<br>+	**β-Hydroxy-D,L-Butyric Acid**<br>No Growth	**α -Keto-Butyric Acid**<br>+	**Acetoacetic Acid**<br>++	**Propionic Acid**<br>+	**Acetic Acid**<br>++	**Formic Acid**<br>++	**Aztreonam**<br>+++	**Sodium Butyrate**<br>++	**Sodium Bromate**<br>No Growth
