# Supplementary material for: Elizabethkingia anophelis MSU001 Isolated from Anopheles stephensi: Molecular Characterization and Comparative Genome Analysis
Source: Microorganisms. 2024 May 27;12(6):1079. doi: 10.3390/microorganisms12061079 (PMC11206156; doi:10.3390/microorganisms12061079)
Supplement: Supplementary file 1 [file microorganisms-12-01079-s001.zip › Table S2 ANI .pdf]

Table S2 average nucleotide identity values (up, black font) and Digital DNA-DNA Hybridization values (low, red font) amongst different *Elizabethkingia* species

		As1	Ag1	R26	AR4_6	AR6_8	MSU001	LDVH AR107	OSUVM 2	CSID 3000521207	JUNP-353	F3201	296-96	SUE	JM-87	NCTC 10016	G4120	FL160902
E. anophelis	**As1**																	
	**Ag1**	99.96																
		99.60																
	**R26**	99.95	99.98															
		99.60	100															
	**AR4-6**	99.96	100	99.99														
		99.60	100	100														
	**AR6-8**	99.96	100	99.99	100													
		99.60	100	100	100													
	**MSU001**	99.96	99.99	99.98	99.99	99.99												
		99.70	100	100	100	100												
	**LDVH-AR107**	97.82	97.91	98.00	97.91	97.91	97.86											
		82.40	82.20	82.80	82.20	82.20	82.20											
	**OSUVM 2**	97.61	97.60	97.53	97.60	97.60	97.60	97.60										
		78.60	78.50	78.50	78.50	78.50	78.50	78.00										
	**CSID 3000521207**	98.41	98.32	98.44	98.32	98.32	98.43	98.19	97.69									
		85.50	86.10	86.10	86.10	86.10	86.00	84.20	79.30									
	**JUNP 353**	98.02	98.00	98.08	98.00	98.00	97.98	97.93	97.52	98.19								
		83.90	84.10	84.60	84.20	84.20	77.00	82.70	78.60	83.30								
	**F3201**	97.45	97.59	97.56	97.59	97.59	97.59	97.56	98.98	97.54	97.51							
		77.70	78.20	78.20	78.20	78.20	78.20	78.20	91.20	79.00	79.10							
	**296-96**	98.51	98.37	98.51	98.37	98.37	98.35	97.74	97.75	98.71	98.02	97.53						
		85.30	85.90	85.90	85.90	85.90	85.90	82.20	79.10	89.30	83.70	78.70						
	**SUE**	98.53	98.38	98.52	98.39	98.39	98.31	97.74	97.73	98.69	98.00	97.55	99.96					
		85.30	85.90	85.90	85.90	85.90	85.90	82.20	79.10	89.20	83.70	78.70	99.80					
	**JM-87**	97.42	97.42	97.39	97.42	97.42	97.42	97.55	98.99	97.61	97.44	98.95	97.53	97.57				
		76.70	76.80	89.30	76.80	76.80	76.80	78.00	91.20	79.60	78.90	91.50	79.00	79.00				
E. meningoseptica	**NCTC10016**	80.41	80.34	80.26	80.34	80.34	80.37	80.36	80.13	80.38	80.26	80.29	80.29	80.26	80.53			
		23.70	23.70	23.70	23.70	23.70	23.60	23.70	23.60	23.80	23.80	24.00	23.60	23.60	24.00			
	**G4120**	80.41	80.28	80.29	80.27	80.27	80.22	80.30	80.39	80.45	80.37	80.25	80.39	80.41	80.28	98.45		
		23.70	23.70	23.70	23.70	23.70	23.60	23.70	32.70	23.80	23.70	23.70	23.60	23.70	23.60	85.80		
E. miricola	**FL160902**	92.16	92.14	92.20	92.14	92.14	92.10	92.11	91.83	92.02	92.10	91.85	92.14	92.17	91.73	80.26	80.34	
		46.60	46.60	46.60	46.60	46.60	46.50	46.50	45.60	46.60	46.30	45.40	46.80	46.80	45.30	23.90	23.80	
