# Supplementary material for: Elizabethkingia anophelis MSU001 Isolated from Anopheles stephensi: Molecular Characterization and Comparative Genome Analysis
Source: Microorganisms. 2024 May 27;12(6):1079. doi: 10.3390/microorganisms12061079 (PMC11206156; doi:10.3390/microorganisms12061079)
Supplement: Supplementary file 1 [file microorganisms-12-01079-s001.zip › Table S4 Total Resistome.pdf]

Table S4 Resistome analysis of *Elizabethkingia* spp.

		E. anopheelis														*E. meningoseptica*		*E.miricola*
Antibiotics	Genes	AR4-6	AR6-8	Ag1	As1	R26	OSUVM2	LDVH AR107	JM-87	CSID 3000521207	F3201	JUNP 353	296-96	SUE	756	NCTC10016	G4120	FL160902
Aminoglycoside	AAC(6')-IIb	-	-	-	-	-	-	-	-	-	-	+	-	-	-	-	-	-
	aadS	+	+	+	+	+	+	+	+	+	+	+	+	+	+	+	+	+
	RanA	+	+	+	+	+	+	+	+	+	+	+	+	+	+	+	+	+
	RanB	+	+	+	+	+	+	+	+	+	+	+	+	+	+	-	+	+
Beta-lactam	BlaB	+	+	+	+	+	+	+	+	+	+	+	+	+	+	-	+	+
	CGA-1	-	-	-	-	-	-	-	-	+	+	+	+	+	-	-	-	-
	CME-1	+	+	+	-	+	+	+	+	+	+	+	+	+	+	+	+	+
	GOB-1	-	-	-	-	-	-	-	+	-	+	-	-	-	-	-	-	-
	GOB-10	-	-	-	-	-	-	+	-	-	-	+	-	-	-	-	-	-
	GOB-11	-	-	-	-	-	-	-	-	-	-	-	-	-	-		-	+
	GOB-16	-	-	-	-	-	-	-	-	-	-	-	+	+	-	+	+	-
	GOB-18	-	-	-	-	-	+	-	-	-	-	-	-	-	-	-	-	-
	GOB-9	+	+	+	+	+	-	-	-	+	-	-	+	+	+	-	-	-
	IND-7	-	-	-	-	-	-	-	-	-	-	-	-	-	+	-	-	-
	OmpA	-	-	-	-	-	-	-	-	-	-	-	-	-	-	+	-	-
	OXA-347	-	-	-	-	-	-	-	-	-	-	+	-	-	-	-	-	-
	TLA-1	+	+	+	+	+	+	+	-	-	+	-	-	-	+	+	+	-
	VEB-7	-	-	-	-	-	-	-	+	-	-	-	-	-	-	-	-	+
diaminopyrimidine	dfrA3	-	-	-	-	-	-	-	-	-	-	-	-	-	-	+	+	-
Elfamycin	EF-Tu	+	+	+	+	+	+	+	+	+	+	+	+	+	+	+	+	+
Fluoroquinolone	gyrA	-	-	-	-	-	-	-	-	-	-	+	+	+	-	-	-	-
	gyrB	+	+	+	+	+	+	+	+	+	+	+	+	+	+	+	+	+
Fusidane	fusE	+	+	+	+	+	+	+	+	+	+	+	+	+	+	+	+	+
glycopeptide	vanXYL	-	-	-	-	-	-	-	-	-	-	-	-	-	-	+	+	-
Isoniazid	katG	+	+	+	+	+	+	+	+	+	+	+	+	+	+	-	+	+
Macrolide	macB-1	+	+	+	+	+	+	+	+	+	+	+	+	+	+	+	-	+
	macB-3	-	-	-	-	-	-	-	-	-	-	-	-	-	-	-	-	+
	mefC	+	+	+	+	+	+	+	+	+	+	+	+	+	+	-	-	+
	macB	-	-	-	-	-	-	-	-	-	-	-	-	-	-	-	+	+
nucleoside	sta	-	-	-	-	-	-	-	-	-	-	-	-	-	-	-	-	+
Para-aminosalicylic acid (PAS)	thyA	+	+	+	+	+	+	+	+	+	+	+	+	+	+	+	+	+
Peptide	bacA	+	+	+	+	+	+	+	+	+	+	+	+	+	+	+	+	+
	bcrA	-	-	-	-	-	-	-	-	-	-	-	-	-	-	-	-	-
	bcrC	-	-	-	-	-	-	-	-	-	-	-	-	-	-	-	-	-
	rosA	-	-	-	-	-	-	-	-	-	-	-	-	-	-	+	-	-
Phenicol	cat	-	-	-	-	-	+	-	-	-	-	-	-	-	-	+	-	-
	catB11	+	+	+	+	+	+	+	+	+	+	+	+	+	+	+	+	+
	catB3	-	-	-	-	-	-	-	-	-	-	+	+	+	-	-	+	-
	catB8	-	-	-	-	-	-	-	-	-	-	+	-	-	-	-	-	-
	AFCA	-	-	-	-	-	-	-	-	-	-	-	-	-	-	+	-	+
Pleuromutilin	TaeA	+	+	+	+	+	+	+	+	+	+	+	+	+	+	+	+	+
Rifamycin	arr-1	-	-	-	-	-	-	-	-	-	+	+	-	-	-	-	-	-
Rifamycin	rpoB	+	+	+	+	+	+	+	+	+	+	+	+	+	+	+	+	+
Streptogramin	vatA	+	+	+	+	+	+	+	+	+	+	+	+	+	+	-	+	+
	vatC	-	-	-	-	-	-	-	+	-	+	-	-	-	-	-	-	-
	vatH	-	-	-	-	-	-	-	+	-	+	-	-	-	-	-	-	-
streptogramin	vatB	-	-	-	-	-	-	-	-	-	-	-	-	-	-	+	-	-
	vatD	-	-	-	-	-	-	-	-	-	-	-	-	-	-	-	-	-
Sulfonamide	sul2	-	-	-	-	-	-	-	-	-	-	+	-	-	-	+	-	-
Tetracycline	otr(A)-1	+	+	+	+	+	+	+	+	+	+	+	+	+	+	-	+	+

	otr(A)-2	-	-	-	-	-	-	-	-	-	-	-	-	-	-	-	-	-
	Tet(X6)	+	+	+	+	+	-	+	-	-	-	+	-	-	+	-	-	+
	TxR-1	+	+	+	+	+	-	+	+	+	+	+	+	+	+	+	+	+
	TxR-2	+	+	+	+	+	-	+	+	+	+	+	+	+	-	+	+	+
	TxR-3	+	+	+	+	+	+	+	-	+	+	+	+	+	+	-	+	+
	TxR-4	-	-	-	-	-	+	-	-	-	-	-	-	-	+	+	-	-
	tet(C)	-	-	-	-	-	-	-	-	-	-	-	-	-	-	-	+	-
	tetB(60)	-	-	-	-	-	-	-	-	-	-	-	-	-	-	-	-	+
Efflux pump	abeS	+	+	+	-	+	+	+	+	+	+	+	+	+	+	+	+	+
	acrR-1	+	+	+	+	+	+	+	+	+	+	+	+	+	+	+	+	-
	acrR-2	+	+	+	+	+	+	-	+	+	+	+	+	+	+	+	-	-
	acrR-3	-	-	-	-	-	-	+	-	-	-	-	-	-	-	-	-	-
	adeB	-	-	-	-	-	-	+	-	-	-	-	-	-	-	-	-	-
	adeF-1	+	+	+	+	+	+	+	+	+	+	+	+	+	+	+	+	+
	adeF-2	+	+	+	+	+	+	+	+	+	+	+	+	+	+	+	+	+
	adeF-3	+	+	+	+	+	+	+	+	+	+	+	+	+	+	+	+	+
	adeF-4	-	-	-	-	-	-	-	-	-	-	-	-	-	-	-	-	-
	adeN	-	-	-	-	-	-	-	-	+	-	-	-	-	-	-	-	-
	arlR-1	-	-	-	-	-	+	-	+	-	-	-	-	-	-	+	+	+
	arlR-2	-	-	-	-	-	-	-	+	-	-	-	-	-	-	-	-	+
	arlR-3	-	-	-	-	-	+	-	+	-	-	-	-	-	-	-	-	-
	arlR-4	-	-	-	-	-	-	-	+	-	-	-	-	-	-	-	-	-
	ErmB	-	-	-	-	-	-	-	-	-	-	-	-	-	-	-	-	-
	ErmF	-	-	-	-	-	-	-	-	-	-	+	-	-	-	-	-	-
	gadW	+	+	+	-	+	+	-	-	-	+	-	-	-	+	-	-	-
	MexL	-	-	-	-	-	-	-	+	-	-	-	-	-	-	+	-	+
	qacH	-	-	-	-	-	-	-	-	-	-	+	-	-	-	+	-	-
	sdiA-1	+	+	+	+	+	+	+	+	+	+	+	+	+	+	+	+	+
	sdiA-2	+	+	+	-	+	-	-	-	-	-	-	-	-	+	-	-	+
	sdiA-3	-	-	-	-	-	-	-	-	-	-	-	-	-	-	-	-	-
	Tet(X3)	-	-	-	-	-	+	-	+	+	+	-	-	-	-	-	-	-
	Tet(X4)	-	-	-	-	-	-	-	-	-	-	-	+	+	-	-	-	-
	tetX	-	-	-	-	-	-	-	-	-	-	+	-	-	-	-	-	-
	ykkC	+	+	+	-	+	+	+	+	+	+	+	+	+	+	-	-	+
	ykkD	+	+	+	+	+	+	+	+	+	+	+	+	+	+	-	-	+
	ceoB	-	-	-	-	-	-	-	-	-	-	-	-	-	-	-	-	-
	MexF	-	-	-	-	-	-	-	-	-	-	-	-	-	-	-	-	-
	smeE	-	-	-	-	-	-	-	-	-	-	-	-	-	-	-	-	-
	ErmB	-	-	-	-	-	-	-	-	-	-	-	-	-	-	-	-	-
